# Microscopic and Molecular Identification of *Sarcocystis* spp. in Intestines of Canids and Mustelids Associated with Sarcocyst-Forming Species in Rodent Muscles

**DOI:** 10.3390/biology15080593

**Published:** 2026-04-08

**Authors:** Adomas Ragauskas, Tamara Kalashnikova, Dovilė Laisvūnė Bagdonaitė, Evelina Juozaitytė-Ngugu, Dalius Butkauskas, Petras Prakas

**Affiliations:** State Scientific Research Institute Nature Research Centre, Akademijos Str. 2, 08412 Vilnius, Lithuania; tamara.kalashnikova@gamtc.lt (T.K.); dovile.bagdonaite@gamtc.lt (D.L.B.); evelina.ngugu@gamtc.lt (E.J.-N.); dalius.butkauskas@gamtc.lt (D.B.); petras.prakas@gamtc.lt (P.P.)

**Keywords:** *Sarcocystis*, genetic identification, *28S* rRNA, definitive host, epidemiology, host interactions, Canidae, Mustelidae, Rodentia

## Abstract

*Sarcocystis*, a diverse and species-rich protist genus infecting reptiles, birds, and mammals, remains poorly understood in terms of true diversity and their lifecycles. Typically, sarcocysts are found in the muscle tissue of the intermediate host (IH), while oocysts undergo sporulation in the intestines of the definitive host (DH). Rodent-associated *Sarcocystis* species often form cryptic species complexes with strong specificity to their DHs; however, their presence in the intestines of wild carnivores, whose IHs are rodents, is understudied. The aim of this study was to investigate the distribution of rodent-associated *Sarcocystis* species in the intestines of wild Mustelidae and Canidae from Lithuania using light microscopy (LM) and nested PCR targeting *28S* rRNA. LM analysis of intestinal scraping revealed *Sarcocystis* spp. in 56.3% of canids and mustelids, while DNA sequence analysis identified 41.0% of mustelids and 11.6% of canids as positive. Three *Sarcocystis* species, *S. arvalis*, *S. myodes*, and *S. ratti*, and the genetic lineage *Sarcocystis* sp. Rod8, which belong to the same cryptic species complex, were identified in mustelids, while *S. arvalis* and *S. myodes* were detected in canids. Thus, mustelids contribute more than canids to the natural transmission of *Sarcocystis* spp. from rodents in Lithuania.

## Simple Summary

Research on associations between parasites and their hosts under natural ecological conditions is significantly lacking. Although studying the distribution of *Sarcocystis* protists is technically demanding and requires both microscopy and molecular tools, such investigations are necessary to address important gaps in the knowledge about their diversity and transmission. In Lithuania, members of the Mustelidae and Canidae families are abundant carnivorous mammals that frequently prey on rodents and are therefore likely important in the transmission of *Sarcocystis* species that use rodents as intermediate hosts. Although these predators are known to host various pathogens, their role in the transmission of rodent-associated *Sarcocystis* species remains poorly studied worldwide. In this study, *Sarcocystis* oocysts and sporocysts were detected microscopically in the intestines of mustelids and canids, while species identification was based on molecular analysis. Examination of the intestines of 151 predatory mammals from Lithuania revealed three *Sarcocystis* species, *S. arvalis*, *S. myodes*, and *S. ratti*, and a genetically distinct lineage, *Sarcocystis* sp. Rod8, that may represent a previously undescribed taxon. Based on DNA sequence analysis, mustelids contribute more than canids to the transmission of rodent-associated *Sarcocystis* spp. in Lithuania, highlighting the complexity of parasitic lifecycles and the need for further regional research.

## 1. Introduction

Members of the genus *Sarcocystis* (Apicomplexa: Sarcocystidae), a species-rich group of coccidian parasites, infect reptiles, birds, and mammals worldwide. To date, over 220 species have been recorded [1,2]; however, the actual number of species is thought to be considerably higher due to limited research [3,4]. *Sarcocystis* spp. are characterized by an obligate heteroxenous two-host lifecycle based on prey–predator relationships involving the formation of sarcocysts in the extra-intestinal tissues, mainly in the muscles, of the intermediate host (IH) and the endogenous sporulation of oocysts in the definitive host (DH) [1,5,6]. Notably, the DH becomes infected through ingestion of animal tissues harbouring mature sarcocysts, whereas the IH acquires infection via contaminated water or food with sporocysts [1,6]. *Sarcocystis* spp. are described in IHs using combined morphological and molecular analyses. In the case of natural infections, parasite species in DHs can generally be differentiated only by molecular analysis, due to common co-infections with multiple *Sarcocystis* spp. and the lack of clear morphological differences between distinct species [3,7,8,9,10]. The pathogenicity of *Sarcocystis* spp. primarily occurs in the IH, whereas infections in the DH are generally asymptomatic. In most cases, *Sarcocystis* spp. cause subclinical infections; however, some species can induce acute disease in livestock, synanthropic, and wild animals [1,6,11].

Of the *Sarcocystis* spp. described to date, predatory mammals are the predominantly confirmed DHs, while reptiles and birds are less commonly identified to serve as DHs [1]. Carnivorous mammals act as DHs of numerous avian and mammalian *Sarcocystis* spp., including those parasitizing economically important livestock and poultry species, as well as wildlife birds and mammals of various families. For instance, most of the *Sarcocystis* spp. identified in cattle, sheep, and Cervidae ungulates are transmittable via predatory mammals [1,12,13]. Historically, DHs have been identified through experimental infections; however, ethical and practical limitations have shifted research toward molecular detection in naturally infected animals. The number of studies using wild carnivores as experimental DHs has substantially decreased due to stricter ethical regulations [12,13,14,15,16]. Moreover, experimental infections do not necessarily confirm that the same host species act as DHs under natural conditions. Additionally, frequent occurrence of mixed-species infections in IH muscles can lead to misinterpretation of experimental results [11,17]. Progress in molecular methods has led to an increasing number of studies identifying *Sarcocystis* spp. directly from intestinal or faecal samples using different genetic markers, allowing for more accurate and ethically acceptable identification of host–parasite associations in DHs under natural conditions [8,9,10,18]. The most commonly used markers were *18S* and *28S* ribosomal RNA (rRNA), internal transcribed spacer 1 (*ITS1*) and mitochondrial cytochrome c oxidase subunit I (*cox1*) [9,10,18,19]. The selection of genetic regions depends on the taxonomic group of IHs, as different markers are recommended for identifying *Sarcocystis* spp., depending on whether their IHs are ungulates, rodents, or birds [20,21,22,23].

The order Carnivora consists of 245 terrestrial species worldwide [24]. Large wild carnivores and mesocarnivores (a carnivorous animal for which 50–70% of its diet is the flesh or meat of another animal) [25] play an important role in regulating ecosystems [26,27,28,29]. Both native and invasive wild mammalian predators are a potential risk factor for transmission of zoonotic pathogens of various origins (e.g., viruses, bacteria, protists, helminths) [30,31] to domesticated animals and humans. This is a crucial challenge for the realization of anthropocentric plans based on the concept of One Health, which is of growing significance and emphasizes the interdependence of human, animal, and environmental health [32]. Notably, the parasites inhabiting these animals require more comprehensive continuous practical and theoretical research due to the limited current knowledge. Around 15 species of wild carnivores inhabit Lithuania [33]. The principal carnivores and mesocarnivores in the country belong to the families Canidae and Mustelidae [34,35]. In Lithuania, the major canids are the carnivore grey wolf (*Canis lupus*), the invasive raccoon dog (*Nyctereutes procyonoides*), and the red fox (*Vulpes vulpes*) [36]. In total, eight mustelid species occur in Lithuania [33]. Among terrestrial mustelids, the beech marten (*Martes foina*), pine marten (*Martes martes*), European badger (*Meles meles*), European polecat (*Mustela putorius*), and American mink (*Neovison vison*) are the most abundant [33,34].

In Lithuania, previous studies on Mustelidae and Canidae as potential DHs have primarily focused on *Sarcocystis* spp., whose IHs are livestock, cervids, or birds [10,19,37,38]. Recently, two new *Sarcocystis* species, *S. myodes* and *S. arvalis*, were identified in bank voles (*Clethrionomys glareolus*) and common voles (*Microtus arvalis*) from Lithuania, and phylogenetic analyses indicated that both species most likely utilize carnivorous mammals as DHs [21,39]. Furthermore, *S. putorii*, a species that utilizes mustelids as DHs, has been reported in Lithuanian rodents [40]. To date, no molecular studies worldwide have investigated the presence of *Sarcocystis* spp. in the intestines of wild carnivores, for which rodents serve as IHs. In this study, the aim was to identify and evaluate the distribution of rodent-associated *Sarcocystis* spp. in the intestines of wild Mustelidae and Canidae from Lithuania using *28S* rRNA sequence analysis accompanied by microscopy results.

## 2. Materials and Methods

### 2.1. Sample Collection and Processing

**Figure 1:**
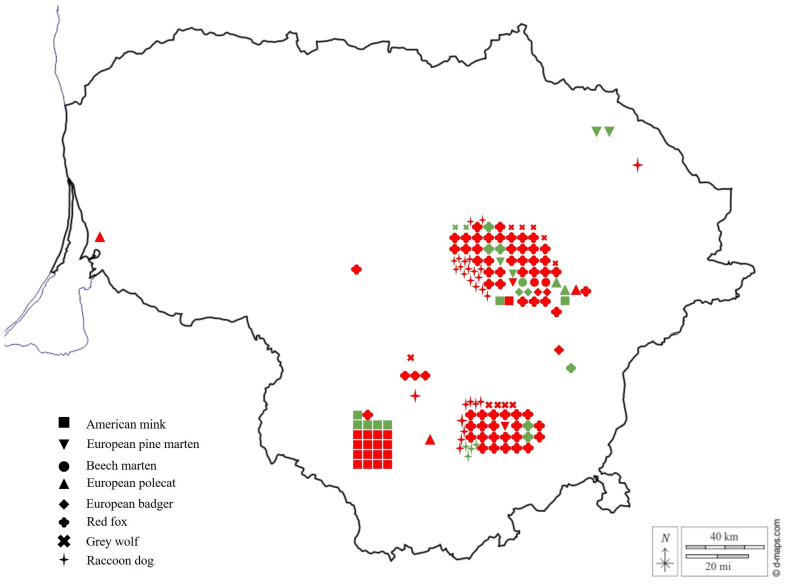
Sampling locations of animals analysed in the current study. Red indicates samples that tested negative during molecular analysis, and green indicates samples positive for rodent-associated *Sarcocystis* spp.

In the present study, intestines of 39 animals belonging to the family Mustelidae and 112 individuals belonging to the family Canidae were screened for *Sarcocystis* spp. Specifically, Mustelidae samples consisted of three beech martens, five European badgers, six pine martens, five European polecats, and 20 American minks, whereas Canidae samples comprised 12 grey wolves, 31 raccoon dogs and 69 red foxes (Figure 1). The examination of animal material was conducted in accordance with the guidelines of the Ethics Committee of the State Scientific Research Institute Nature Research Centre (no. GGT-1; issued on 11 January 2024).

The Minister of Environment in Lithuania approves the rules on hunting (20 June 2002, no. IX-966) of the Republic of Lithuania, sets a list of game species, and defines limits on the time and means of hunting. Under the legislation, hunting of invasive American minks, raccoon dogs as well as red foxes is permitted throughout the year in Lithuania. Hunting of beech martens, pine martens and European polecats are allowed from 1 July to 1 April, whereas European badgers can be legally hunted from 1 October to 1 December. According to Council Directive 92/43/EEC on the Conservation of Natural Habitats and of Wild Fauna and Flora, the grey wolf is a protected species throughout the European Union. In Lithuania, grey wolves can be hunted from 15 October to 1 April, with seasonal quotas set by the Minister of Environment. The animals of listed species were legally hunted mainly in the eastern, southern, and central parts of Lithuania in the 2021–2025 period. All animals included in this study came from legally hunted individuals during the regular annual hunting season; no animals were killed for the purpose of this research. In cooperation with local hunters, intestine samples of grey wolves and some raccoon dogs as well as red foxes were delivered to the Laboratory of Molecular Ecology of State Scientific Research Institute Nature Research Centre, Vilnius, Lithuania. Meanwhile, full carcasses of mustelids and some raccoon dogs, as well as red foxes, were provided to the laboratory. Carcasses of red foxes and other predator species were frozen—at −20 °C for a minimum of 90 days before examination to ensure inactivation of *Echinococcus* sp. eggs, in accordance with recommended biosafety procedures.

### 2.2. Microscopical Analysis of Sarcocystis spp.

The small intestine was removed from animals and cut lengthwise. The intestinal epithelium was gently scraped with a scalpel, and the material was suspended in 100 mL of distilled water. The isolation of oocysts and sporocysts of *Sarcocystis* spp. was conducted following previously established methods [41]. The modifications included in the processing of intestinal material are described in detail in the previous work [42]. After processing, samples were examined for oocysts and sporocysts of *Sarcocystis* spp. under a light microscope (LM) at ×400 magnification. Notably, DNA was extracted from all samples, regardless of whether *Sarcocystis* sp. stages were observed under an LM.

### 2.3. Molecular Examination of Sarcocystis spp.

DNA was extracted with the GeneJET Genomic DNA Purification Kit (Thermo Fisher Scientific Baltics, Vilnius, Lithuania) following the manufacturer’s protocol. Purified genomic DNA was stored at −20 °C for further molecular manipulation. *Sarcocystis* spp. were identified using nested PCR (nPCR) targeting *28S* rRNA and the subsequent Sanger sequencing of amplified products. Two external primers (Sgrau281 5′-GAACAGGGAAGAGCTCAAAGTG-3′ and Sgrau282 5′-GGTTTCCCCTGACTTCATTCTAC-3′) amplifying about a 900 bp fragment were used in the first step of nPCR [43], whereas in the second step of nPCR, a single forward primer, SgrauzinF 5′-CCTGTGTCATTTAGTTCCACGTA-3′, was applied with a combination of three alternative reverse primers: SmyodesR 5′-TAAAAAGAAAAGTTCCAACGGTGT-3′, SrattiR 5′-CCAGAATCCTTTCACCCCAAC-3′ and SspRod1R 5′-CTGGAGTCTTTTCGTCCCAAC-3′. Specifically, SmyodesR, SrattiR and SspRod1R were in silico designed using Primer 3 plus software [44] to amplify *S. myodes*, *S. arvalis* and *Sarcocystis* spp. using rodents and carnivores as their IHs and DHs, respectively. The primer pair SgrauzinF/SmyodesR theoretically amplifies a 420 bp fragment, whereas SgrauzinF/SrattiR and SgrauzinF/SspRod1R amplify a 357 bp fragment. Nuclease-free water was used as the negative control in both nPCR steps. Positive controls included DNA of *S. myodes*, *S. arvalis* or *S. meriones*, which was isolated from individual sarcocysts and identified by sequencing in previous investigations [21,39,45].

Reactions of nPCR were carried out in a 25 µL volume using DreamTaq PCR Master Mix (Thermo Fisher Scientific Baltics, Vilnius, Lithuania). For the first step of nPCR, 12.5 µL of PCR mix, 0.5 µM of each primer, 4 µL of extracted DNA and 7.5 µL of nuclease-free water were used. The composition of the second step was the same as in the first step, except that 2 µL from the first nPCR product was used instead of template DNA. The PCR thermal cycling conditions were as follows: initial denaturation at 95 °C for 5 min, followed by 35 cycles consisting of denaturation at 94 °C for 35 s, annealing at 58–63 °C depending on the primer pair for 45 s, and elongation at 72 °C for 55 s, concluding with a final extension step at 72 °C for 5 min.

The amplified products were visualized and evaluated using 1% agarose gel electrophoresis. Amplicons of the correct size without non-specific bands were enzymatically purified with exonuclease ExoI and alkaline phosphatase FastAP (Thermo Fisher Scientific Baltics, Vilnius, Lithuania). All amplified target samples of the second step of nPCR were bidirectionally sequenced using the Big-Dye^®^ Terminator v3.1 Cycle Sequencing Kit (Thermo Fisher Scientific, Vilnius, Lithuania) and the 3500 Genetic Analyzer (Applied Biosystems, Foster City, CA, USA). The *28S* rRNA sequences obtained in the present study were deposited in NCBI GenBank with PX804151–PX804203 accession numbers.

### 2.4. Data Analysis

In the current work, the generated *28S* rRNA sequences were compared with those of *Sarcocystis* spp. using the Nucleotide BLAST online tool [46] (https://blast.ncbi.nlm.nih.gov/, accessed on 19 December 2025). Phylogenetic analyses were conducted using MEGA 12.0.14 software [47]. Sequences were aligned with the help of the ClustalW algorithm, and the Maximum Likelihood (ML) method was used for phylogenetic inference. The best fitting nucleotide substitution model for the analysed datasets was chosen based on the lowest values of the Bayesian Information Criterion (BIC), calculated using the “Find Best DNA/Protein Models (ML)” option. *Hyaloklossia lieberkuenhi*, *Eumonospora henryae* and *Cystoisospora yuensis* were set as the outgroup. The reliability of the phylogenetic tree was assessed using the bootstrap method with 1000 replicates. The ratio of transitions to transversions (*Ts*/*Tv*) was established to evaluate substitution biases in datasets.

A 95% confidence interval (CI) for the prevalence of *Sarcocystis* in each DH species and family, as well as for the frequency of co-infections, was calculated using Sterne’s exact method [48]. Pair-wise comparisons of prevalence were computed by the unconditional exact test [49], while Fisher’s exact test was employed for analyses of datasets comprising more than two samples. All statistical analyses were conducted in Quantitative Parasitology 3.0 software [50], and differences were considered statistically significant at *p* < 0.05.

## 3. Results

### 3.1. Detection of Sarcocystis sp. Oocysts and Sporocysts by Light Microscopy

**Figure 2:**
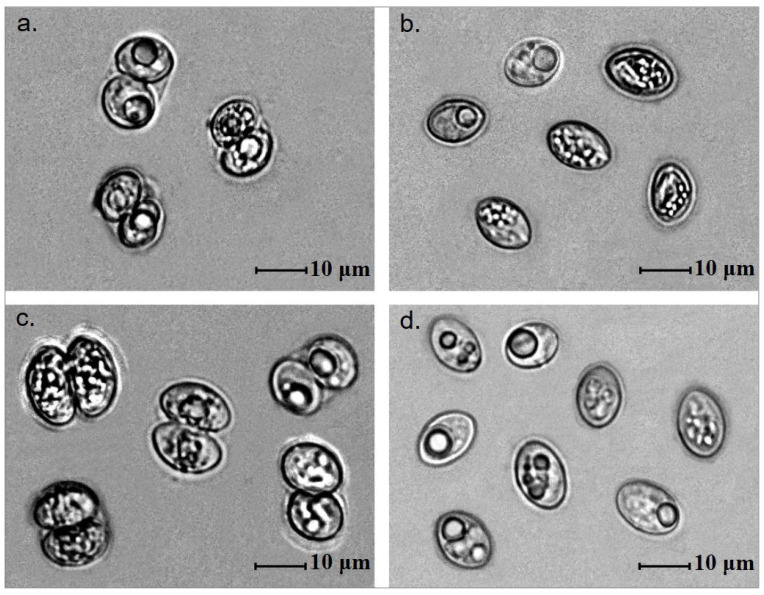
Oocysts/sporocysts found in small intestine mucosal scrapings of *Canidae* and *Mustelidae* species. (**a**,**c**) Sporulated oocysts. (**b**,**d**) Sporocysts. *Sarcocystis* spp. from pine marten (**a**,**b**) and red fox (**c**,**d**).

*Sarcocystis* sp. sporocysts and/or sporulated oocysts were observed in the intestinal epithelia of the small intestines of all eight species of canids and mustelids analysed in the current study (Figure 2). Free sporocysts were seen more frequently than sporulated oocysts. The highest number of *Sarcocystis* sp. parasites, i.e., 320 sporocysts, were observed in the 24 × 24 mm coverslip of mucosal scrapings from one grey wolf and a single red fox collected in the Trakai and Ukmergė districts, respectively.

LM analysis of intestinal scraping samples detected *Sarcocystis* sp. infection in 85 out of 151 (56.3%) canids and mustelids. Specifically, sporocysts or oocysts of the parasite were found in 33.3% (13/39) of mustelids and 64.3% of canids (72/112). The observed prevalence of *Sarcocystis* spp. by LM was significantly higher in canids than mustelids (*p* = 0.0008). No unsporulated oocysts were detected in the investigated species. The average size of sporulated oocysts of *Sarcocystis* spp. in mucosal scrapings of mustelids measured 8.3–22.4 × 12.3–23.9 μm (13.2 ± 3.6 × 16.9 ± 3.0 μm; *n* = 75) (Figure 2a), whereas that of free sporocysts was 6.4–11.5 × 7.0–17.5 μm (8.3 ± 0.8 × 12.4 ± 1.4 μm; *n* = 160) (Figure 2b). Meanwhile, sporulated oocysts of *Sarcocystis* spp. in canids measured 8.1–19.7 × 13.6–28.7 μm (14.0 ± 2.3 × 19.8 ± 2.7 μm; *n* = 240) (Figure 2c), while free sporocysts were 7.1–13.9 × 10.1–20.8 μm (9.8 ± 1.0 × 14.0 ± 1.7 μm; *n* = 569) (Figure 2d). Therefore, the sporocyst and sporulated oocyst sizes found in different species of canids and mustelids overlapped (Table 1); however, on average, canid oocysts and sporocysts appeared to be larger than those collected from mustelids. According to animal species, the largest average sporocyst size was observed in grey wolves (10.5 × 16.2), while the smallest average was found in beech marten (7.8 × 10.2). Meanwhile, on average, American minks exhibited the largest sporulated oocysts (18.3 × 21.1), whereas European pine marten had the smallest (9.3 × 14.6).

### 3.2. Genetic Identification of Sarcocystis Species

Overall, 53 sequences of *28S* rRNA were obtained (Table 2). Similar numbers of sequences, i.e., 26 and 21, were generated with the SgrauzinF/SspRod1R and SgrauzinF/SmyodesR primers, respectively. By contrast, only six samples were amplified with the SgrauzinF/SrattiR primer pair. Overall, 23 sequences were assigned to *S. arvalis* (PX804151–PX804173), 18 to *S. myodes* (PX804174–PX804191) and one to *S. ratti* (PX804192). Finally, five identical 313 bp sequences (PX804193–PX804197) and six identical 373 bp sequences (PX804198–PX804203) generated with the SgrauzinF/SspRod1R and SgrauzinF/SmyodesR primers, respectively, exhibited 100% identity in the overlapping region. Notably, these sequences differed by at least 1% from all other *Sarcocystis* sp. sequences. Specifically, the longer 373 bp sequences of this parasitic taxon shared 97.9–98.1% similarity with those of *S. arvalis*, 96.8–97.9% similarity with those of *S. myodes*, 96.8% similarity with those of *S. meriones*, and 96.3% similarity with those of *S. ratti*. Therefore, 11 sequences of this genetic lineage were tentatively assigned to *Sarcocystis* sp. Rod8, as our research group previously identified seven other *Sarcocystis* spp. whose sequences exhibited the highest similarities to sequences obtained from sarcocysts in rodents.

**Figure 3:**
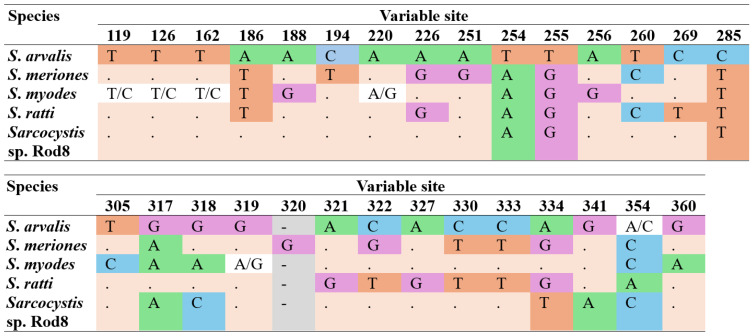
Variable sites in *28S* rRNA sequences of closely related *S. arvalis*, *S. meriones*, *S. myodes*, and *Sarcocystis* sp. Rod8. Dashes indicate deletion.

Comparisons of the numbers of substitutions across the *Sarcocystis* sp. Rod8 lineage and closely related *S. arvalis*, *S. meriones*, *S. myodes* and *S. ratti* were made (Figure 3). *Sarcocystis* sp. Rod8 showed the lowest genetic divergence from *S. arvalis* (with seven single-nucleotide polymorphisms (SNPs), including three transitions). It differed from *S. myodes* by eight SNPs, including five transitions, from *S. meriones* by 11 SNPs, including seven transitions and one insertion, and from *S. ratti* by 14 SNPs, including 10 transitions. Among the analysed *Sarcocystis* spp., the lowest divergence was observed between *S. ratti* and *S. meriones*, with seven differences, whereas the largest divergence (17 SNPs) was found between *S. myodes* and *S. ratti*. The proportion of transitions relative to transversions (*Ts*/*Tv*) increased with evolutionary distance, reflecting the higher prevalence of transitions in this conserved *28S* rRNA region.

**Figure 4:**
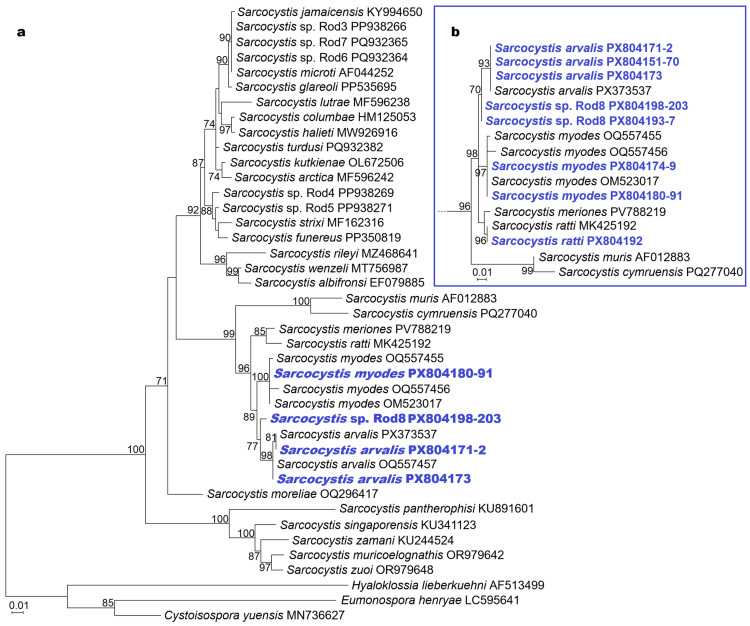
Phylogenetic relationships of *Sarcocystis* spp. identified in intestines of canids and mustelids examined. Phylogenetic trees were constructed using 373 bp (**a**) and 313 bp (**b**) *28S* rRNA fragments obtained in present study. The final multiple sequence alignments comprised 41 unique sequences and 411 nucleotide positions, including gaps, for (**a**), and 44 unique sequences and 328 nucleotide positions, including gaps, for (**b**). In panel (**b**), only a partial phylogram containing a cluster with sequences determined in this work is displayed. Phylograms were generated using the ML method, scaled according to branch length and rooted on *H. lieberkuenhi*, *E. henryae* and *C. yuensis*. Kimura 2-parameter and HKY+G evolutionary models were set for (**a**) and (**b**) analyses. Sequences obtained in the present work are displayed in indigo colour. Figures next to branches show bootstrap support values, and GenBank accession numbers are provided after species names.

Based on *28S* rRNA sequences, the lineage *Sarcocystis* sp. Rod8 clustered within a rodent-associated *Sarcocystis* sp. complex comprising *S. meriones*, *S. ratti*, *S. arvalis*, and *S. myodes*. Two additional species, *S. cymruensis* and *S. muris*, which also use rodents as IHs, formed a sister clade to this complex, characterized by short branch lengths. *Sarcocystis* sp. Rod8 was the most closely related to *S. arvalis*, while *S. myodes* was sister to this clade, and *S. meriones* clustered with *S. ratti* (Figure 4). Notably, sequences of *S. arvalis*, *S. myodes* and *S. ratti* acquired in this study were placed with high support together with sequences of certain species. Furthermore, two different clades of *S. arvalis* were identified, as two sequences obtained in this study grouped with *S. arvalis* from common vole (*M. arvalis*) (PX373537), while a single sequence grouped with *S. arvalis* from tundra vole (*Alexandromys oeconomus*) (OQ557457). Across the five discussed closely related *Sarcocystis* spp., the overall *Ts*/*Tv* ratio was 2.77, reflecting a predominance of transitions, whereas among *Sarcocystis* Rod8 and its closest relatives (*S. arvalis* and *S. myodes*), the *Ts*/*Tv* ratio was lower at 1.50, indicating a relatively balanced accumulation of nucleotide substitutions.

### 3.3. Distribution of Detected Sarcocystis Species Across Mustelids and Canids Examined

*Sarcocystis* spp. were detected in all five Mustelidae and all three Canidae species examined (Table 3). However, due to the low sample sizes for beech martens, European badgers, European pine martens, and European polecats (three to six individuals each), meaningful conclusions regarding differences in the *Sarcocystis* prevalence among these host species cannot be drawn. No significant differences in the detection rates of *Sarcocystis* spp. were observed among the five Mustelidae species (*p* = 0.749). Likewise, comparison of prevalence between the grey wolf, the raccoon dog and the red fox revealed no significant differences (*p* = 0.744). In contrast, the detection rate of *Sarcocystis* spp. in 112 canids (11.6%) was significantly lower (*p* = 0.0001) than that recorded in 39 mustelids (41.0%).

All four identified *Sarcocystis* spp. were found in the European polecat, while in other DHs, one or two parasite species were confirmed by molecular analysis. Notably, four *Sarcocystis* spp. were established in mustelids and only two species, *S. arvalis* with *S. myodes*, were confirmed in the three canid species.

Co-infections with two different *Sarcocystis* spp. were detected in five individual animals. Specifically, *S. arvalis* and *S. myodes* co-infections were identified in a single beech marten and a single red fox, whereas *S. arvalis* together with lineage *Sarcocystis* sp. Rod8 was detected in one American mink. In addition, two European polecats harboured mixed infections, comprising *S. arvalis* with *S. myodes* in one individual and *S. ratti* with lineage *Sarcocystis* sp. Rod8 in another (Table 3). Overall, the prevalence of co-infections was significantly higher (*p* = 0.0098) in mustelids (10.3%) than in canids (0.9%), whereas no significant interspecific differences in the *Sarcocystis* co-infection rates were observed within the Mustelidae or Canidae families (*p* = 0.090 and *p* = 1.000, respectively).

Among the targeted rodent-associated *Sarcocystis* spp., *S. arvalis* was the most frequently detected, followed by *S. myodes*, indicating that these two species are the dominant parasites in the intestines of the examined carnivores (Table 4). Lineage *Sarcocystis* sp. Rod8 was less common, and *S. ratti* was identified only in a single European polecat. No significant differences were observed in the distribution of *Sarcocystis* spp. among Mustelidae (*p* = 0.114), in contrast to the uneven distribution of species in Canidae (*p* < 0.001). Two *Sarcocystis* spp. found in both carnivore families, *S. arvalis* and *S. myodes*, were more frequently detected in Mustelidae than in Canidae; however, these differences were not statistically significant (*p* = 0.0565 in both cases).

## 4. Discussion

### 4.1. Prevalence of Sarcocystis spp. in Mustelids and Canids

Epidemiological studies on the role of wild mustelids as DHs in the natural transmission of *Sarcocystis* spp. remain notably scarce compared to similar examinations involving canids. Besides investigations carried out by our research group in 2021–2025 on the prevalence of *Sarcocystis* spp. in intestinal scrapings of mustelids [19,37,51,52,53], other research involving these predators has not been conducted. Previously, microscopical examination of intestinal samples of one to five species of mustelids (*n* = 20–115) revealed prevalence of *Sarcocystis* spp. in two neighbouring countries, Lithuania and Latvia, ranging from 37.5% to 70.0% [52,53]. In the present study, LM analysis revealed a 33.3% prevalence of *Sarcocystis* parasites in mustelids. The relatively low infection rate in the current study can be explained by the bias toward American mink samples (*n* = 20) compared to other mustelids (*n* = 19). While 25.0% of the invasive American mink were positive for *Sarcocystis* spp., the combined prevalence in all other host species was 42.1%. Similarly, higher *Sarcocystis* infection rates were observed in other mustelid species compared to American mink (56.8% vs. 37.5%) in one of our previous studies [37]. Therefore, *Sarcocystis* sp. infection rates likely vary by mustelid species; however, low sample sizes for some species require further studies to confirm these findings.

In contrast to mustelids, epidemiological studies on the role of wild canids in the natural transmission of *Sarcocystis* spp. appear to be broader in terms of geographic scope (Europe, Asia, North America, South America), study period (1982–2026), and type of biological material used (faecal or intestinal samples) [3,7,10,38,54,55,56,57,58]. Notably, red foxes, raccoon dogs, and grey wolves have been the most commonly investigated species for *Sarcocystis* spp., as in the current study. The prevalence of *Sarcocystis* spp. in these studies, as determined by morphological methods, varied greatly from 2.8% in the intestines of Slovenian red foxes [59] to 92.3% in the intestines of Lithuanian grey wolves [10]. In several studies, both the intestines and faeces of the same canid species were examined, and in all cases, *Sarcocystis* spp. were more prevalent in intestinal samples [7,54,60]. The current work revealed a relatively high (64.3%) prevalence of *Sarcocystis* spp. in intestinal scrapings of canids. Differences in detection rates of *Sarcocystis* spp. in canids may be linked to host dietary preferences, geographical regions, host age, seasonal patterns, and methodological peculiarities for sample handling, preparation, and examination [7,38,54,61].

It is worth noting that occurrence rates of *Sarcocystis* spp. detected by microscopic analysis and molecular methods cannot be directly compared, as the current molecular methods used are biased towards specific groups of IHs [3,37,51]. This study focused only on *Sarcocystis* spp. associated with rodents. Therefore, samples that were positive for *Sarcocystis* spp. via LM analysis but were negative by PCR likely harbour *Sarcocystis* spp. utilizing other types of IHs, such as ungulates or birds. To understand the full scope of *Sarcocystis* spp. utilizing mustelids and canids as DHs, larger-scale molecular studies are needed.

### 4.2. Genetic Identification of Sarcocystis spp. Associated with Rodents in Intestines of Mustelidae and Canidae

Recent molecular studies suggest that *S. arvalis*, *S. meriones*, *S. myodes* and *S. ratti* represent a cryptic species complex associated with a rodent–mammal lifecycle. These species can be discriminated by *28S* rRNA and *ITS1*; however, the later region is difficult to amplify and sequence [39,45]. These *Sarcocystis* spp. utilize rodents of the genera *Alexandromys*, *Apodemus*, *Clethrionomys*, *Microtus*, *Meriones* and *Rattus* as IHs and, based on phylogenetic data, carnivorous mammals as DHs [39,45,62]. Current data indicates that lineage *Sarcocystis* sp. Rod8 is part of this *Sarcocystis* sp. complex. Notably, other cryptic *Sarcocystis* sp. complexes associated with rodents can be distinguished. Broadly discussed, the *Sarcocystis*-*zuoi* complex comprises species with small mammal–snake lifecycles. Members of this complex, including *S. attenuati*, *S. kani*, *S. muricoelognathis*, *S. scandentiborneensis*, and *Sarcocystis zuoi*, use rodents, specifically rats of the genera *Ratus* and *Maxomys*, shrews or treeshrews as IHs and colubrid snakes as DHs [63,64,65]. Members of this complex can be reliably distinguished using *18S* rRNA and *ITS1*, rather than *cox1*. Moreover, the currently available phylogenetic evidence points to the possible existence of an additional species complex with a rodent–bird lifecycle, including *S. glareoli*, *S. microti*, *S. jamaicensis*, *Sarcocystis* sp. Rod3, *Sarcocystis* sp. Rod6 and *Sarcocystis* sp. Rod7 [2,17,66,67]. These species can be confidently distinguished through *28S* rRNA and *ITS1* analyses [9,66].

Despite early descriptions of *Sarcocystis* spp. that use rodents or other small mammals as IHs, this parasite group remains significantly understudied [1]. A major limitation is the low prevalence rate of *Sarcocystis* spp. in IHs [68,69]. Additionally, some *Sarcocystis* spp. that form microscopic sarcocysts are morphologically undistinguishable under LM and require transmission electron microscopy (TEM) and molecular analysis [21,22,70]. Furthermore, a major part of *Sarcocystis* spp. from rodents are characterized only morphologically [71,72], lacking molecular data entirely [43,63,64]. In cases where molecular data is available, some of it is outdated and insufficient, as new studies have revealed the limited suitability of previously used genetic markers [21,22,64,70]. One of the indications of the lack of comprehensive morphological and molecular studies on *Sarcocystis* spp. infecting rodents is the fact that, since 2023, our research group has molecularly detected eight distinct rodent-associated *Sarcocystis* lineages in biological samples from Lithuania and Spain. Seven of these lineages (*Sarcocystis* sp. Rod1–Rod7) were identified in our earlier studies [2,9,43,67], whereas an additional lineage, *Sarcocystis* sp. Rod8, was detected for the first time in the present study. None of these lineages could be genetically assigned to any previously described *Sarcocystis* spp. at the time of their detection. Recently, *S. arvalis* was described in the common vole, and its molecular identity was demonstrated to correspond to *Sarcocystis* sp. Rod1 [39]. Beyond the studies discussed above, there is a growing number of reports documenting the presence of rodent-associated *Sarcocystis* spp., which nevertheless remain unidentified at the species level [65,70,73,74,75].

### 4.3. The Transmission of Rodent-Associated Sarcocystis Species

Approximately 50 *Sarcocystis* spp. have been described in rodents, of which data on partial DNA is available for only one-third of the species [39,45,63,76]. Historically, DHs were determined through lifecycle experiments and confirmed reptiles, birds, and mammals as DHs of rodent-associated *Sarcocystis* spp. [63,64,76,77]. The role of carnivorous mammals in transmitting *Sarcocystis* spp. from rodents is poorly understood. To date, a limited subset of species has been confirmed to use mammalian DHs from the families Canidae, Felidae, and Mustelidae. Some *Sarcocystis* spp. exhibit broader host associations and have been reported in both Felidae and Mustelidae (e.g., *S. muris*, *S. putorii*), or in Canidae and Mustelidae (e.g., *S. undulati*) [1,78]. However, no species have been conclusively demonstrated to complete their lifecycle in both Canidae and Felidae. Other species appear to be more host-specific: *S. baibacinacanis* and *S. rhombomys* are associated with canids, *S. cymruensis* and *S. neotomafelis* with felids, and *S. campestris* with mustelids [1,78]. The results of the present study indicate a tendency toward a higher occurrence of *S. arvalis* and *S. myodes* in Lithuanian mustelids; however, these two species can presumably be transmitted by both canids and mustelids, whereas lineage *Sarcocystis* sp. Rod8 was detected exclusively in mustelids. In contrast, *S. ratti* was found in only a single sample, precluding any reliable conclusions regarding its DH range.

### 4.4. The Underestimated Role of Mustelids in the Transmission of Sarcocystis Species

It is generally accepted that *Sarcocystis* spp. transmitted via canids cannot be transmitted via felids, and vice versa [1]. An exception to this pattern is *S. wenzeli*, which parasitizes chickens and has been shown to use both dogs and cats as DHs. As compared to canids and felids, mustelids are less commonly examined as potential DHs of *Sarcocystis* spp. [37]. This pattern may reflect the fact that transmission experiments have predominantly focused on dogs and cats, given their frequent contact with farm animals, which are the most thoroughly examined for *Sarcocystis* spp. In general, studies examining naturally infected mustelids and canids, or mustelids and felids together, for their role in the transmission of various *Sarcocystis* spp. are lacking. Recently, molecular analyses indicated that canid-associated *Sarcocystis* spp. (e.g., *S. arieticanis*, *S. bertrami*, *S. capracanis*, *S. cruzi*) or felid-associated *Sarcocystis* spp. (*S. bovifelis*, *S. hirsuta*) can be detected in intestines of mustelids [37,51]. Furthermore, cervid-associated *Sarcocystis* spp., which previously were shown by experimental studies not to be transmitted via canids or felids, were confirmed in mustelids through DNA sequence analysis [52,53]. Furthermore, mustelids were demonstrated to be involved in the transmission of *Sarcocystis* forming sarcocysts in birds [19,53]. Finally, the present study indicates that mustelids may be involved in the transmission of four *Sarcocystis* spp. from rodents. Based on previous studies and current results, the role of mustelids in *Sarcocystis* transmission is significant and has been previously underestimated.

### 4.5. Comparison of Mustelidae and Canidae in Transmission of Rodent-Associated Sarcocystis Species

This study revealed that both the prevalence of *Sarcocystis* spp. associated with rodents and the proportion of co-infections were higher in Mustelidae than in Canidae, with four species detected in mustelids compared to two in canids (Table 3 and Table 4). Based on data presented in the literature, we assume that this pattern is driven more by the phylogeny and taxonomy of DHs than by distribution, abundance, or feeding ecology [33,34,35,79,80,81,82,83].

The majority of the studied animals, except the grey wolf, were mesocarnivores, [36,79], yet the *Sarcocystis* sp. prevalence and species richness in the studied canids was lower compared to those in mustelids. Dietary differences provide only a partial explanation for the observed patterns. Currently, among mustelids, only the diet of the European pine marten has been comprehensively studied in Lithuania [80]. The European pine marten feeds extensively on small mammals, including voles (*Clethrionomys* spp., *Microtus* spp.) and mice (*Apodemus* spp.) [80], and shows substantial dietary overlap with the red fox (62.4–96.5% shared rodent prey) [81]. In contrast, raccoon dogs consume considerably fewer rodents (10.5–13.3%) than red foxes (40.1–40.9%) and pine martens (38.9–46.0%) [82], while grey wolves primarily prey on larger ungulates [83]. Overall, both mustelids and canids in Lithuania consume small mammals, but the higher *Sarcocystis* sp. prevalence in mustelids suggests either greater exposure or higher susceptibility to *Sarcocystis* sp. infection; however, further studies are needed to clarify these patterns.

### 4.6. Limitations, Implications, and Future Perspectives

Despite providing novel insights into the transmission of rodent-associated *Sarcocystis* spp., this study has several limitations. Only the *28S* rRNA gene was used for molecular identification, which limits the resolution of species discrimination [9,39,63,70]. In addition, a relatively small primer set was employed, further restricting the ability to detect different *Sarcocystis* spp. Although additional genetic markers could improve accuracy, their amplification and sequencing from naturally infected carnivores remain technically challenging [21,39,43,70]. Furthermore, developing suitable genetic markers and primers for rodent–carnivore *Sarcocystis* spp. remains difficult due to the limited molecular data available for this group [21,22,39,45]. Finally, the number of animals examined per carnivore species was relatively limited, which could have reduced the detection rate of rare species or co-infections.

Nevertheless, this study represents the first large-scale investigation of rodent-associated *Sarcocystis* across two carnivore families, Mustelidae and Canidae. Furthermore, it provides critical insights into host associations and transmission patterns. The current results emphasize the underrecognized role of mustelids in the transmission of several *Sarcocystis* species, complementing the existing knowledge that has largely focused on canids and felids.

Future research should focus on multi-host studies in areas where multiple carnivore families coexist. Such studies would allow for a more comprehensive assessment of the relative contributions of different DHs to *Sarcocystis* sp. transmission. Additionally, using a broader set of molecular markers and standardized detection methods across studies will improve species identification and facilitate the comparison of the prevalence, host specificity, and potential effects of *Sarcocystis* parasites on wildlife and domestic animals. Attention should also be directed toward comprehensive research on the studied *Sarcocystis* cryptic species complex, including detailed investigations across different continents to assess the number of *Sarcocystis* spp. within the species complex. Finally, genetic and ecological relationships of different *Sarcocystis* cryptic species complexes involving rodents as IHs in the same geographic areas should be investigated.

## 5. Conclusions

This study provides the first large-scale molecular investigation of rodent-associated *Sarcocystis* spp. in the intestines of Mustelidae and Canidae. Four closely related species, *S. arvalis*, *S. myodes*, *S. ratti* and the genetically new lineage *Sarcocystis* sp. Rod8, were identified by *28S* rRNA sequence analysis. These species together with *S. meriones* form a cryptic species complex. Based on the species richness, prevalence, and co-infection rates, mustelids appear to contribute more to the natural transmission of rodent-associated *Sarcocystis* spp. in Lithuania than canids. The findings underscore the ecological complexity of rodent–carnivore *Sarcocystis* lifecycles and highlight the need for further studies in the same geographic regions, involving different carnivore families and additional molecular markers.

## Figures and Tables

**Table 1:** Detection rates and morphologies of oocysts/sporocysts found in small intestine mucosal scrapings of Mustelidae and Canidae species from Lithuania.

Animal	Infected/Investigated (%) by Microscopical Detection of *Sarcocystis* spp.	Infected/Investigated (%) by Molecular Detection of *Sarcocystis* spp.	Sporocysts (μm)	Sporulated Oocysts (μm)
American mink	5/20 (25.0%)	7/20 (35.0%)	7.8–8.8 × 10.2–12.7 (8.3 ± 0.3 × 11.9 ± 0.7, *n* = 32)	13.5–22.4 × 14.8–23.5 (18.3 ± 2.9 × 21.1 ± 2.5; *n* = 14)
Beech marten	3/3 (100%)	1/3 (33.3%)	7.0–8.6 × 7.0–12.6 (7.8 ± 0.6 × 10.2 ± 1.7; *n* = 10)	10.1–17.5 × 13.5–23.9 (13.1 ± 2.4 × 16.2 ± 2.7; *n* = 23)
European badger	1/5 (20.0%)	2/5 (40.0%)	6.4–9.6 × 10.0–14.1 (8.1 ± 0.8 × 12.7 ± 1.0; *n* = 25)	9.7–16.0 × 13.5–18.6 (13.1 ± 2.0 × 16.2 ± 1.7; *n* = 17)
European pine marten	3/6 (50.0%)	4/6 (66.7%)	7.4–11.5 × 10.5–17.5 (9.0 ± 1.1 × 13.5 ± 2.2, *n* = 21)	8.3–10.6 × 12.3–16.2 (9.3 ± 0.8 × 14.6 ± 1.3, *n* = 15)
European polecat	1/5 (20.0%)	2/5 (40.0%)	6.7–9.5 × 10.5–14.6 (8.3 ± 0.7 ×12.5 ± 0.8, *n* = 72)	10.4–12.8 × 16.0–18.4 (11.5 ± 0.8 × 17.6 ± 0.7, *n* = 6)
Grey wolf	10/12 (83.3%)	2/12 (16.7%)	8.2–13.9 × 11.6–20.8 (10.5 ± 1.2 × 16.2 ± 1.5, *n* = 140)	10.9–19.7 × 16.1–28.7 (15.7 ± 1.9 × 20.2 ± 2.6, *n* = 78)
Raccoon dog	15/31 (48.4%)	4/31 (12.9%)	7.1–12.6 × 10.7–18.2 (9.8 ± 1.0 × 14.0 ± 1.5, *n* = 229)	10.1–19.0 × 13.6–26.6 (13.8 ± 2.0 × 18.6 ± 2.7, *n* = 32)
Red fox	47/69 (68.1%)	7/69 (10.1%)	7.5–10.1 × 12.8–18.8 (9.5 ± 0.9 × 13.4 ± 1.3, *n* = 200)	8.1–14.7 × 18.6–27.0 (13.0 ± 2.1 × 19.4 ± 2.6, *n* = 130)

**Table 2:** Genetic identification of *Sarcocystis* spp. in intestines of mustelids and canids from Lithuania based on *28S* rRNA sequences.

Species	GenBank Acc. No.	Primers	Length	Genetic Similarity Comparing	
				with Same Species	with Most Closely Related Species
*S. arvalis*	PX804151–PX804155	SgrauzinF/SrattiR	313	100%	96.8–97.8% *S. myodes*; 97.8% *S. ratti*; 97.4% *S. meriones*; 93.3% *S. moreliae*
*S. arvalis*	PX804156–PX804170	SgrauzinF/SspRod1R	313	100%	96.8–97.8% *S. myodes*; 97.8% *S. ratti*; 97.4% *S. meriones*; 93.3% *S. moreliae*
*S. arvalis*	PX804171–PX804172	SgrauzinF/SmyodesR	373	99.7–100%	96.0–97.1% *S. myodes*; 96.5% *S. ratti*; 96.3% *S. meriones*; 91.0% *S. cymruensis*
*S. arvalis*	PX804173	SgrauzinF/SmyodesR	373	99.7–100%	96.3–97.3% *S. myodes*; 96.5% *S. meriones*; 96.3% *S. ratti*; 90.9% *S. cymruensis*
*S. myodes*	PX804174–PX804179	SgrauzinF/SspRod1R	313	99.0–100%	98.1% *S. ratti*; 97.8% *S. arvalis*, 97.8% *S. meriones*; 93.3% *S. moreliae*
*S. myodes*	PX804180–PX804191	SgrauzinF/SmyodesR	373	98.9–100%	97.1–97.3% *S. arvalis*; 96.3% *S. meriones*; 95.7% *S. ratti*; 91.4% *S. moreliae*
*S. ratti*	PX804192	SgrauzinF/SrattiR	313	100%	99.0% *S. meriones*; 97.1–98.1% *S. myodes*; 97.8% *S. arvalis*; 93.6% *S. muris*
*Sarcocystis* sp. Rod8	PX804193–PX804197	SgrauzinF/SspRod1R	313	-	99.0% *S. arvalis*; 97.8–98.7% *S. myodes*; 98.7% *S. ratti*; 98.4% *S. meriones*; 94.0% *S. moreliae*
*Sarcocystis* sp. Rod8	PX804198–PX804203	SgrauzinF/SmyodesR	373	-	97.9–98.1% *S. arvalis*; 96.8–97.9% *S. myodes*; 96.8% *S. meriones*; 96.3% *S. ratti*; 91.7% *S. moreliae*

**Table 3:** Prevalences and proportions of *Sarcocystis* co-infections in intestines of five Mustelidae and three Canidae carnivore species in Lithuania.

Host Species or Family	N	Infected	Prevalence (95% Confidence Intervals)	*Sarcocystis* Species	Proportion of Co-Infections (95% Confidence Intervals)
American mink	20	7	35.0 (16.7–57.6)	*S. arvalis*, *Sarcocystis* sp. Rod8	5.0 (0.3–24.4)
Beech marten	3	1	33.3 (1.7–86.5)	*S. arvalis*, *S. myodes*	33.3 (1.7–86.5)
European badger	5	2	40.0 (7.7–81.1)	*S. arvalis*	0
European pine marten	6	4	66.7 (27.1–93.7)	*S. myodes*	0
European polecat	5	2	40.0 (7.7–81.1)	*S. arvalis*, *S. myodes*, *S. ratti*, *Sarcocystis* sp. Rod8	40.0 * (7.7–81.1)
Mustelidae	39	16	41.0 (26.7–57.8)	*S. arvalis*, *S. myodes*, *S. ratti*, *Sarcocystis* sp. Rod8	10.3 (3.6–24.1)
Grey wolf	12	2	16.7 (3.1–45.7)	*S. arvalis*, *S. myodes*	0
Raccoon dog	31	4	12.9 (4.5–28.8)	*S. arvalis*, *S. myodes*	0
Red fox	69	7	10.1 (4.9–19.4)	*S. arvalis*, *S. myodes*	1.4 (0.1–7.7)
Canidae	112	13	11.6 (6.5–19.1)	*S. arvalis*, *S. myodes*	0.9 (0.1–4.8)

* In one animal, *S. arvalis* + *S. myodes* Rod8 co-infections were identified, while in another animal, *S. ratti* + *Sarcocystis* sp. Rod8 co-infections were identified.

**Table 4:** Prevalences of four identified *Sarcocystis* species in mustelids and canids examined.

Sarcocystis Species	Number of Animals Infected	Prevalence in Mustelidae (95% Confidence Intervals)	Prevalence in Canidae (95% Confidence Intervals)
*S. arvalis*	15	18.0 (8.6–33.2)	7.1 (3.4–13.7)
*S. myodes*	12	15.4 (6.9–30.4)	5.4 (2.4–11.5)
*S. ratti*	1	2.6 (0.1–13.6)	0
*Sarcocystis* sp. Rod8	6	15.4 (6.9–30.4)	0

## Data Availability

The *28S* rRNA sequences of *S. arvalis*, *S. myodes*, *S. ratti*, and *Sarcocystis* sp. Rod8 obtained in the present study were deposited in NCBI GenBank under accession numbers PX804151–PX804203.
